# Gene Methylation and Silencing of WIF1 Is a Frequent Genetic Abnormality in Mantle Cell Lymphoma

**DOI:** 10.3390/ijms22020893

**Published:** 2021-01-18

**Authors:** Abdulraheem Alshareef, Anthea C. Peters, Pascal Gélébart, Will Chen, Raymond Lai

**Affiliations:** 1 Medical Laboratories Technology Department, College of Applied Medical Sciences, Taibah University, Madinah, P.O. Box 41477, Saudi Arabia; amshareef@taibahu.edu.sa; 2 Department of Laboratory Medicine and Pathology, University of Alberta, Edmonton, AB T6G 2E1, Canada; pascal.gelebart@uib.no (P.G.); will.chen@ualberta.ca (W.C.); 3 Department of Medicine, University of Alberta, Edmonton, AB T6G 2E1, Canada; anthea1@ualberta.ca; 4 Department of Clinical Science, University of Bergen, 5021 Bergen, Norway; 5 Department of Oncology, University of Alberta, Edmonton, AB T6G 2E1, Canada

**Keywords:** mantle cell lymphoma, Wnt canonical pathway, Wnt inhibitory factor-1, gene methylation

## Abstract

We have previously shown that the Wnt canonical pathway (WCP) is constitutively active in most cases of mantle cell lymphoma (MCL). Here, we aimed to elucidate the mechanisms underlying this biochemical deregulation. We hypothesized that gene methylation/silencing of WIF1 (Wnt inhibitory factor-1), a physiologic inhibitor of WCP, contributes to the deregulation of WCP and promotes cell growth in MCL. In support of this hypothesis, we found that the expression of WIF1 was detectable in none of the 4 MCL cell lines, and in only 2 of 5 tumors (40%) examined. Using methylation-specific PCR, we found evidence of gene methylation of WIF1 in 4 of 5 cell lines (80%) and in 24 of 29 (82%) tumors. The addition of the demethylation agent 5-aza-2′-deoxycytidine to Mino and JeKo-1, two WIF1-negative cell lines, restored the expression of WIF1 mRNA in these cells. Gene transfection of WIF1 into JeKo-1 and Mino cells significantly reduced cell growth, and this finding correlated with substantial downregulations of various proteins in WCP, such as β-catenin and pGSK-3β. In conclusion, our results support the concept that gene methylation/silencing of WIF1 is a frequent event in MCL, and this abnormality contributes to the aberrant activation of WCP. These results have provided further evidence that aberrant Wnt signaling is pathogenetically important in MCL and it may represent a potential therapeutic target.

## 1. Introduction

Mantle cell lymphoma (MCL) is a type of aggressive B-cell non-Hodgkin lymphoma that carries a guarded prognosis despite a high rate of initial remission induced by conventional chemotherapy regimens [1,2]. Most of these tumors harbor the characteristic chromosomal translocation, t(11;14) (q13;q32), which brings the cyclin D1 gene under the influence of the enhancer of IgH, resulting in aberrant cyclin D1 expression in these neoplastic B-cells [3]. In view of its normal function, cyclin D1 is believed to drive tumorigenesis by promoting cell-cycle progression. Nevertheless, more recent studies have provided evidence that cyclin D1 may promote tumorigenesis via functions unrelated to its cell-cycle regulatory functions [4,5,6]. It is also widely believed that the over-expression of cyclin D1 alone is not sufficient for lymphomagenesis. For example, cyclin D1 transgenic mice do not have spontaneous lymphoma formation. For instance, in the animal model described by Smith et al, MCL-like tumors were induced in Eµ-cyclin D1 transgenic mice only after injection with pristane, a tumor promoter [7]. Thus, oncogenic events additional to the aberrant over-expression of cyclin D1 are likely to be important for the development of MCL.

Constitutive activation of the Wnt canonical pathway (WCP) has been shown to promote tumorigenesis in many types of cancer [8,9,10,11,12,13]. Details of WCP have been reviewed elsewhere [14,15,16]. Normally, activation of WCP is initiated by the binding of various Wnt ligands to their respective cell-surface receptors that are made up of the frizzled family members and the low-density lipoprotein receptor-related protein (LRP)-5 or LRP-6. This event leads to the inactivation of the destruction complex consisting of glycogen synthase kinase (GSK)-3β, axin, and adenomatous polyposis coli (APC), involving the phosphorylation of GSK-3β and the subsequent nuclear accumulation of β-catenin. In complex with the T-cell factor (TCF)/lymphocyte-enhancing factor (LEF) family of transcription factors, β-catenin modulates the transcription of a host of important genes encoding Myc, cyclin D1, and various Wnt agonists. In many types of cancer, WCP is constitutively active, and this aberrancy has been shown to promote cell proliferation and survival. Correlating with this, the activation status of WCP has been shown to significantly correlate with a worse clinical outcome. For example, our group has previously demonstrated that GSK-3β phosphorylation and inactivation, which was found in most cases of MCL examined, significantly correlates with a short survival [17].

The mechanisms underlying the constitutive activation of WCP in MCL have not been extensively studied. We hypothesized that the expression of naturally occurring WCP inhibitors might be defective in these neoplastic B-cells. In this study, we evaluated the expression status of one of these inhibitors, Wnt inhibitory factor-1 (WIF1), which is a secreted protein that normally binds to Wnt ligands and prevents them from activating the WCP.

## 2. Results

### 2.1. Expression of WIF1 in MCL Cell Lines and Patient Tumor Samples

**Figure 1:**
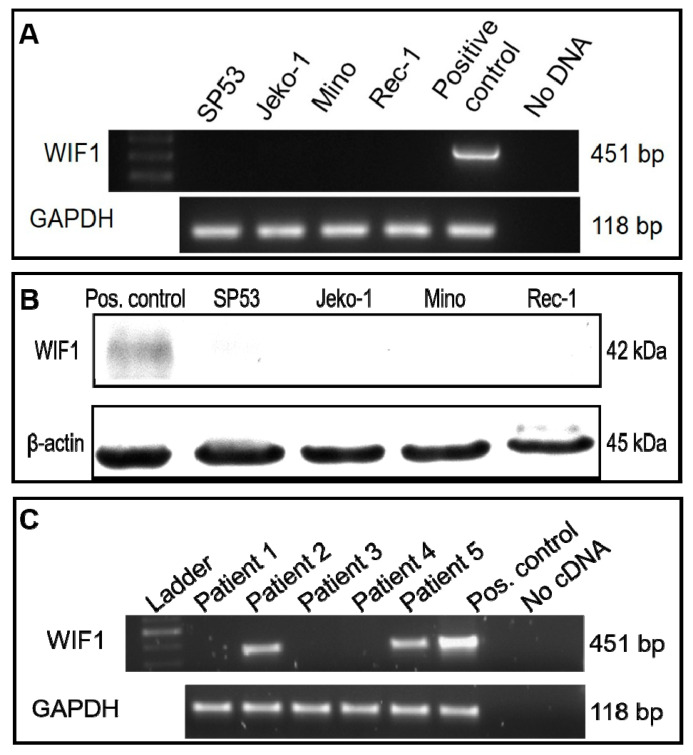
Wnt inhibitory factor-1 (WIF1) mRNA and protein expression were not detected in mantle cell lymphoma (MCL) cell lines and the majority of patient samples. (**A**). WIF1 mRNA expression was not detected in the 4 tested MCL cell lines. (**B**). WIF1 protein expression was not detected in the 4 tested MCL cell lines. (**C**). WIF1 is not expressed in three out of five MCL patient samples. RNA and protein derived from transfected JeKo-1 cells with WIF1 were used as a WIF1 positive control in all experiments.

To examine the expression of WIF1 in MCL, we performed RT-PCR and Western blot studies. As shown in Figure 1A,B, WIF1 mRNA and WIF1 protein were not detectable in all 4 MCL cell lines examined, including SP53, JeKo-1, Mino, and Rec1. Using RT-PCR to detect WIF1 mRNA in 5 cases of MCL tumors from previously untreated patients, WIF1 expression was detected in only 2 out of the 5 (40%) samples (Figure 1C).

### 2.2. WIF1 Is Hypermethylated in Most MCL Cell Lines and Tumors

**Figure 2:**
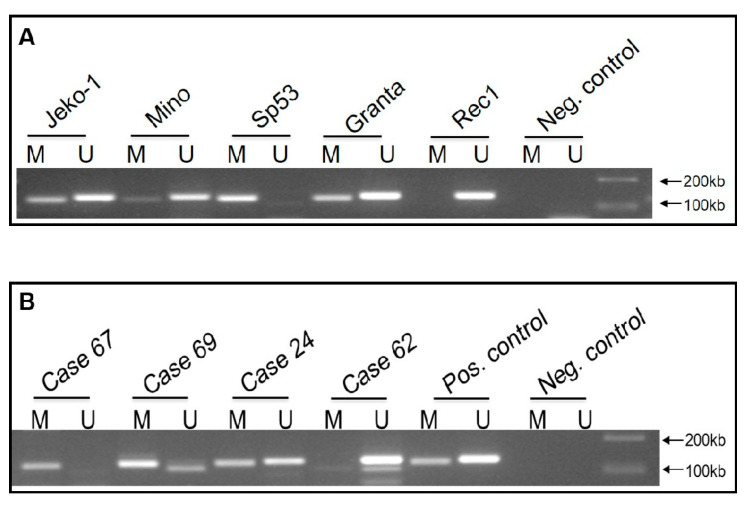
High methylation status of the WIF1 promoter region in MCL cell lines and patients. (**A**) Methylation-specific PCR of the WIF1 promoter region was performed on 5 MCL cell lines. With the exception of REC1, signals from the methylation-specific PCR were readily detectable. (**B**) Methylation-specific PCR of WIF1 promoter region performed on 4 MCL patient samples. M indicates primer specific for methylated site, U indicates primer specific for unmethylated site. Neg indicates no DNA negative control.

We speculated that WIF1 is not expressed in most MCL cell lines and patient samples due to gene methylation and silencing. Thus, we employed methylation-specific PCR. As shown in Figure 2A, 4 out of 5 (80%) MCL cell lines showed amplification with the methylation-specific primer set, with Rec1 being the only cell line showing no detectable evidence of gene methylation. Using the same primer set and PCR condition, we examined the methylation status of the WIF1 promoter in 29 tumor samples from previously untreated MCL patients. Twenty-four out of the 29 (83%) patient samples showed evidence of WIF1 gene methylation (illustrated in Figure 2B).

We have previously published that the expression of phosphorylated/inactivated GSK-3β (pGSK-3β) is detectable in approximately two-thirds of MCL tumors [17]. In this same study, the expression of pGSK-3β was found to significantly correlate with the nuclear expression of β-catenin. Thus, we asked if the methylation status of WIF1 correlates with the expression of pGSK-3β. As shown in Table 1, the methylation status of WIF1 was in alignment with the expression of pGSK-3β in 21 of 29 (72.4%) cases. Specifically, the presence of WIF1 methylation correlates with the expression of pGSK-3β, or vice versa. The overall correlation between these two parameters is statistically significant (*p* = 0.038). A handful of ‘outliers’ were identified. Six (21%) cases had WIF1 methylation that was coupled with a low level of pGSK-3β. Only 2 (6.9%) cases showed a high expression of pGSK-3β in the absence of WIF1 methylation.

### 2.3. WIF1 Expression Is Restored by Treatment with Demethylation Agent 5-aza

**Figure 3:**
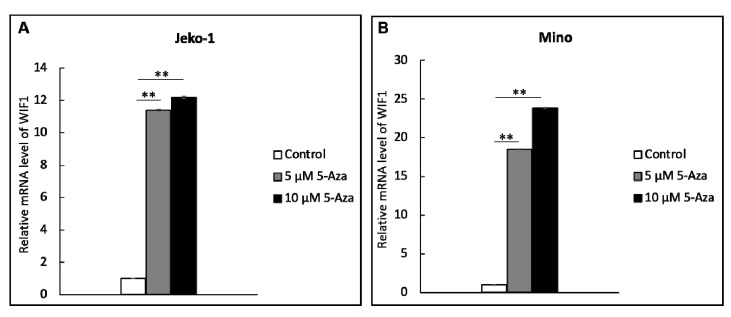
The expression of WIF1 mRNA was dramatically increased in (**A**) JeKo-1 and (**B**) Mino cells after the treatment with the demethylating agent, 5-aza. ** *p* value < 0.001.

We then determined whether gene methylation is directly responsible for the silencing of WIF1 in MCL cells. Thus, we treated JeKo-1 and Mino—both of which showed evidence of WIF1 gene methylation—with 5-aza, a commonly used demethylation agent. As shown in Figure 3, WIF1 mRNA expression in both JeKo-1 and Mino cells significantly increased after the 5-aza treatment.

### 2.4. WIF1 Decreases β-Catenin and pGSK-3β Expression in MCL Cell Lines

**Figure 4:**
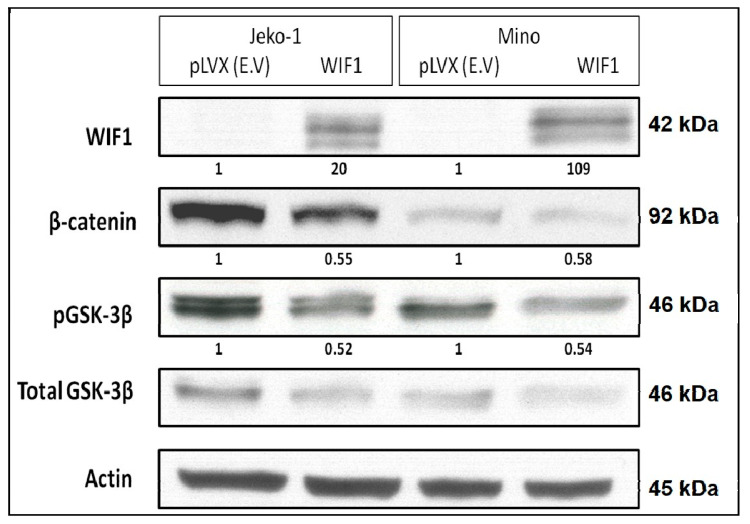
The expression of several markers of the Wnt canonical pathway was down-regulated 48 h after the gene transfection of WIF1. The densitometry readings of individual bands on the Western blot are shown.

*In vitro* experiments were conducted using two MCL cell lines (JeKo-1 and Mino). As shown in Figure 4, we found that gene transfection of WIF1 resulted in more than 45% reduction in the protein levels of β-catenin and pGSK-3β. These findings suggest that the loss of WIF1 expression is indeed an important contributor to the constitutive activation in MCL.

### 2.5. WIF1 Inhibits MCL Cell Growth and Sensitizes Them to Cytarabine (Ara-C)

**Figure 5:**
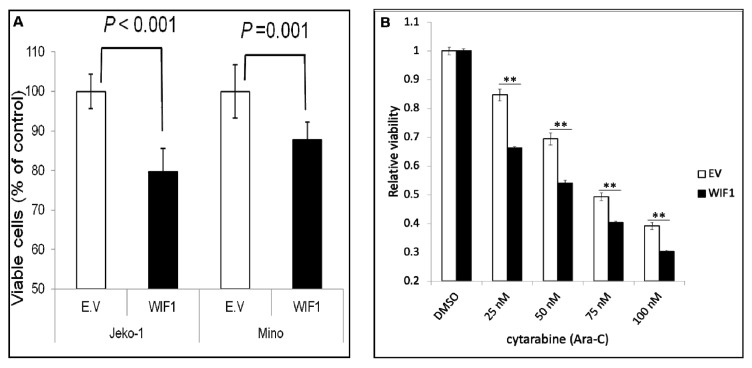
WIF1 inhibits MCL cell growth and sensitizes them to cytarabine (Ara-C). (**A**) Transient gene transfection of WIF1 significantly decreased the number of viable cells. (**B**) The same treatment also significantly increased the inhibitory effect of cytarabine (Ara-C) in JeKo-1 cells. MTS assay was used to assess the number of viable cells. ** *p* value < 0.001.

We then assessed if the restoration of WIF1 by gene transfection can induce any biological changes. As shown in Figure 5A, transfection of WIF1 significantly reduced the cell growth by 20–30% in both JeKo-1 and Mino cells. Triplicate experiments were performed, and results from a representative run are illustrated. Furthermore, WIF1-transfected JeKo-1 cells sensitized these cells to the chemotherapeutic drug cytarabine (Ara-C). Ara-C was added to WIF1-transfected JeKo-1 cells 24 h after the gene transfection, the number of viable cells was quantified using MTS assay 48 h after the addition of Ara-C. As shown in Figure 5B, restoration of WIF1 significantly sensitized JeKo-1 cells to Ara-C-induced inhibition on cell growth.

## 3. Discussion

Despite the recent advances in therapeutics, the overall prognosis of MCL remains guarded and disease relapses are relatively frequent [1,2]. Identification of new therapeutic targets is somewhat limited by incomplete understanding of the biology of this disease. Another confounding factor is that MCL is increasingly recognized to be heterogeneous, both biologically and clinically. Defects in WCP were initially reported by our group [18], in which we employed immunohistochemistry and identified 33 (52%) of 64 MCL tumors showing constitutive WCP activation, as evidenced by the nuclear localization of β-catenin and the expression of the p-GSK3β. In a follow-up study, we found that a high expression of p-GSK3β, which made up 67.5% of our MCL cohort (*n* = 83) correlated with a significantly shorter overall survival [17]. In a subsequent study using gene profiling comparing MCL cells from patient’s peripheral blood samples with normal tonsillar B-cells, a research group identified additional evidence that WCP is deregulated in MCL [19]. Taken together, it appears that deregulations of WCP exist in a subset of MCL tumors and these defects carry prognostic implications in this disease.

DNA methylation is the one of key biological processes under the realm of epigenetic gene regulation. In humans, it occurs on the cytosine residue in the CpG-rich region that is commonly found in the promoter region of many genes. Hypermethylation of various tumor suppressor genes is known to be an important mechanism to contribute to the pathogenesis of many types of cancer [20,21,22,23]. Accordingly, various studies have shown that restoration of the expression of tumor suppressors, using gene transfection or pharmacologic agents (e.g. 5-AZA), has been shown to induce effective apoptosis and cell growth arrest in cancer cells. Demethylation agents have also been clinically used as anti-cancer agents [24,25,26]. Regarding WIF1, our literature search has identified relatively few studies detecting WIF1 methylation in various tumors [27,28,29,30]. This defect has been reported to carry prognostic significance in several types of solid tumors, such as colorectal carcinoma [31] and endometrial carcinoma [32]. While most of these studies focused on the detection of WIF1 methylation in their cohorts of clinical samples and its prognostic value, the biological significance of this defect was not examined in detail. To our knowledge, studies of WIF1 methylation in hematologic malignancies are found only in 5 studies [10,13,33,34,35], to be further discussed below.

In this study, we have shown that WIF1 methylation is a highly frequent event in MCL, amounting to 82% of the tumors included in our study cohort. Furthermore, we have provided direct evidence that this defect is biologically significant, since the restoration of WIF1 expression can downregulate the activation status of WCP, suppress cell growth and sensitize MCL cells to Ara-C-induced growth inhibition. These findings are in parallel with those described in the previous studies in which the biological significance of WIF1 methylation in cancers was examined. Nonetheless, most of these studies of WIF1 methylation in hematological malignancies did not examine the biological significance of this defect. For instance, Chim et al surveyed a cohort of chronic lymphocytic leukemias and found that WIF1 is infrequently methylated in these neoplasms [36]. In another article published by Griffiths et al, WIF1 hypermethylation was detected in 32% of acute myeloid leukemia samples [13]. One of the 5 studies of WIF1 in hematologic malignancies is related to MCL [33]. In this study, it was demonstrated that WIF1 was not detectable in all 4 MCL cell lines examined, although MCL tumors were not included. Functional studies, which involved WIF1 gene transfection, were performed only in a diffuse large B-cell lymphoma cell line (i.e. Pfeiffer). Taken together, results from this current study have shown for the first time that WIF gene methylation/silencing is highly frequent in MCL tumors and we have provided direct evidence that WIF1 gene methylation is biologically significant in MCL.

Due to the relatively high frequency of WIF1 methylation in our MCL cohort, a determination of the prognostic value of this biomarker is rather difficult. Nonetheless, we found a statistical correlation between WIF1 methylation and the high expression of pGSK-3β, and this finding suggests that WIF1 methylation may also have prognostic value. Regarding the ‘outliers’, most of these cases showed evidence of WIF1 methylation that was coupled with a low level of pGSK-3β. One explanation for this apparent mismatch may be related to a failure of the immunohistochemical detection of pGSK-3β, possibly due to suboptimal antigen preservation. Another possible explanation is related to the existence of additional defects (e.g. mutations) in the downstream pathway of WCP, leading to a failure of phosphorylation/inactivation of GSK-3β. Relevant to the second explanation, it has been reported that restoration of WIF1 expression in colorectal cancer cell lines can induce significant apoptosis in these cells, despite the fact that they are known to carry WCP downstream mutations [37]. These observations suggest that WIF1 may exert tumor suppressor functions independent of the downstream WCP pathway. In other words, restoration of WIF1 may be therapeutically useful even in these ‘outlier’ cases, since the WIF1 can mediate tumor suppressor effects via other mechanisms.

In conclusion, we have demonstrated that WIF1 is frequently silenced epigenetically in MCL cell lines and tumors. We have provided direct evidence that this defect is biologically significant in MCL. Our finding of a significant correlation between the expression of pGSK-3β and WIF1 gene methylation has provided some degree of validation that this biochemical defect is likely biologically significant in MCL tumors as well. Further studies may be focused on testing if restoration of WIF1 is a feasible therapeutic approach in treating MCL.

## 4. Materials and Methods

### 4.1. Cell Lines and Tumor Samples

Five previously described MCL cell lines, JeKo-1, Mino, SP53, Granta, and Rec1, were used in this study. Briefly, the five cell lines carry a mature B-cell immunophenotype, they are negative for the Epstein-Barr virus nuclear antigen, positive for cyclin D1 overexpression, and the t(11;14)(q13;q32) cytogenetic abnormality. Cell lines were grown in RPMI 1640 supplemented with 10% fetal bovine serum and glutamine under a 5% CO_2_ atmosphere. All MCL tumors were diagnosed at the Cross Cancer Institute (Edmonton, Alberta, Canada) between 1994 and 2007, and the diagnostic criteria were based on those described in the World Health Organization Classification Scheme [38]. All cases were confirmed to over-express cyclin D1 by immunohistochemistry. The use of these tissues was approved by our institutional ethics committee (approval number Pro00062737; expiry date 25 November 2021).

### 4.2. Reverse Transcription-PCR

Total RNA from MCL cell lines and MCL patient samples was isolated using TRIzol (Invitrogen, Carlsbad, CA, USA) in accordance with the manufacturer’s suggested protocol. Briefly, cDNA synthesis was carried out for 30 min at 42 °C using SuperScript Reverse Transcriptase II (Invitrogen). The PCR was performed for 30 cycles in a thermal cycler (Applied Biosystems, Streetville, Ontario, Canada), with each consisting of denaturation (94 °C for 1 min), primer annealing (58 °C for 1 min), and DNA extension (72 °C for 1.5 min for 30 cycles). Amplified products were electrophoresed in 2% agarose gel containing ethidium bromide and visualized using an Alpha Imager 3400 (Alpha Innotech, San Leandro, CA, USA). Primers for reverse transcription PCR were obtained from Invitrogen (Alameda, CA). A total of 500 ng RNA was used in each reaction. Primer sequences were as follows: forward primer 5′ CCG AAA TGG AGG CTT TTG TA 3′, reverse primer 5′ GTG TCT TCC ATG CCA ACC TT 3′ (product size 451 bp). Glyceraldehyde-3-phosphate dehydrogenase (GAPDH) was used as an internal control.

### 4.3. Methylation Specific PCR (MSP)

DNA was extracted from paraffin-embedded MCL tumor tissue and MCL cell lines using the Qiagen DNeasy Tissue Kit according to the manufacturer’s protocol (Qiagen, Mississauga, Ontario, Canada). Bisulfite-treated genomic DNA was amplified using either a methylation-specific or an unmethylation-specific primer set. HotStarTaq DNA polymerase (Qiagen) was used in the experiments. Sequences of the methylation-specific primers were 5′-GGGCGTTTTATTGGGCGTAT-3′ (forward) and 5′-AAACCAACAATCAACGAAC-3′ (reverse). Sequences of the unmethylation-specific primers were 5′-GGGTGTTTTATTGGGTGTAT-3′ (forward) and 5′-AAACCAACAATCAACAAAAC-3′ (reverse) corresponding to the WIF1 promoter region sequences −488 to −468 and −310 to −290, respectively [39,40].

### 4.4. Treatment with 5-aza-2’-deoxycytidine

5-Aza-2’-deoxycytidine (Sigma-Aldrich, Toronto, Ontario, Canada) was dissolved in 50% acetic acid and diluted to 10 μg/μL and aliquots were stored at −20 °C. Cells were seeded at a density of 0.5 × 10^6^ per mL in 6-well plates. 5-Aza was diluted in fresh RPMI medium and added to the culture medium to final concentrations of 5 and 10 μM every 24 h for 3 days. Sterilized 50% acetic acid at a concentration approximately equal to that used for 5-aza-treated cultures was added to negative control cultures every 24 h for 3 days.

### 4.5. Western Blot Analysis and Antibodies

Western blot analysis was performed using standard techniques. Briefly, the cells were washed with phosphate-buffered saline (PBS), and cellular proteins were precipitated using RIPA buffer containing 150 mM NaCl, 1%NP40, 0.5% deoxycholic acid, 0.1% SDS, 50 mM Tris pH8 which was supplemented with 40.0 µg/mL leupeptin, 1 µM pepstatin, 1 mM 4-(2-aminoethyl)-benzenesulfonyl fluoride (AEBSF) and 0.1 mM phenylmethylsulfonyl-fluoride (PMSF). The protein concentration of the samples was determined using BCA Protein Assay Kit (Pierce, Thermo Fisher Scientific Inc, Rockford, IL, USA). The supernatant was removed and 50 µg of protein was run on an 8%-12% SDS polyacrylamide gel. After the proteins were transferred to nitrocellulose membranes, the membranes were blocked with 5% milk in tris-buffered saline (TBS) buffer (20 mM Tris-HCl, pH 7.6, 150 mM NaCl), and then incubated with primary antibodies overnight at 4 °C temperature followed by 1 h incubation with horseradish peroxidase-conjugated secondary antibody (Jackson Immunoresearch Laboratories, Inc., West Grove, PA, USA). The membranes were washed in PBS with 0.1% Tween-20 for 30 min between steps. Proteins were detected using the Enhanced Chemiluminescence Detection Kit (Amersham Life Sciences, Arlington Heights, IL, USA). Antibodies used were: anti-WIF1 (1:500) and anti-pGSK3β (1:1000, Ser9) (Cell Signaling, Danvers, MA, USA), anti- β-catenin (amino acid residue 571–581, 1:500, BD Transduction laboratories, Lexington, KY, USA), and anti-actin (1:3000, Sigma-Aldrich, St Louis, MO, USA).

### 4.6. Lentiviral Gene Transfection

WIF1 overexpression was assessed using two different methods. (1) The WIF1 expression vector pcDNA3.1 vector was a generous gift from Professor Qian Tao, Cancer Center, Chinese University of Hong Kong. Transient transfections of MCL cells (10 × 10^6^ cells) were performed using the Electro square electroporator (BTX ECM 800, Holliston, MA, USA) (225 V, 8.5 msec, 03 pulse) using 1 μg of WIF1 or empty vector per million MCL cells. Cells were harvested at 48 h after transfection. The efficiency of target gene overexpression was assessed using Western blot. (2) Lentiviral particles were generated by transfecting the 293T packaging cell line (Clontech Laboratories, Inc., Fremont, CA, USA) with the WIF1 lentiviral vector (pLenti-GIII-CMV-Puro) (Applied Biological Materials Inc., Richmond, BC, Canada) or its control (pLVX-Puro lentiviral vector) (Clontech Laboratories, Inc., Fremont, CA, USA), according to the manufacturer’s suggestion. CMV sequencing primer 5′-CGCAAATGGGCGGTAGGCGTG-3′ in which WIF1 sequenced and verified. JeKo-1 and Mino cells were infected with lentiviral particles at 24 and 48 h. Forty-eight hours after the second infection, cells were washed with phosphate-buffered saline (PBS) and lysed using the previous Western blot protocol.

### 4.7. MTS Assay and Cell Viability

MCL cell lines in RPMI 1640 were seeded 48 h post-transfection in a 96-well plate and the assay was conducted following the manufacturer’s instructions (Promega, Nepean, ON, Canada). The measurements were obtained at a wavelength of 450 nM using a Biorad Microplate Reader. The absorbance values were normalized to the untreated cells using the microplate Manager 5.2.1 software (Biorad, Hercules, CA, USA). All experiments were performed in triplicate. Cell viability was determined using the trypan blue exclusion test and results expressed as the total number of viable cells.

### 4.8. Statistical Analysis

Data are expressed as mean +/− standard derivation. Statistical significance was tested using Student’s t-test and statistical significance was achieved when the *p* value was <0.05.

## Figures and Tables

**Table 1:** A significant correlation between the high expression of pGSK-3β and WIF1 methylation in 29 cases of mantle cell lymphoma.

Methylation Status	Low pGSK-3β	High pGSK-3β	*p* Value
WIF1 methylation detected	6	15	0.038
WIF1 methylation NOT detected.	6	2	

## Data Availability

The data presented in this study are available within the article.
